# Nitrate Assimilation in *Fusarium fujikuroi* Is Controlled by Multiple Levels of Regulation

**DOI:** 10.3389/fmicb.2017.00381

**Published:** 2017-03-14

**Authors:** Andreas Pfannmüller, Jana M. Boysen, Bettina Tudzynski

**Affiliations:** Molecular Biology and Biotechnology of Fungi, Department of Biology, Institute of Biology and Biotechnology of Plants, University of MünsterMünster, Germany

**Keywords:** *Fusarium*, nitrate reductase, NirA, AreA, nitrate sensing, nitrogen metabolite repression, gene regulation

## Abstract

Secondary metabolite production of the phytopathogenic ascomycete fungus *Fusarium fujikuroi* is greatly influenced by the availability of nitrogen. While favored nitrogen sources such as glutamine and ammonium are used preferentially, the uptake and utilization of nitrate is subject to a regulatory mechanism called nitrogen metabolite repression (NMR). In *Aspergillus nidulans*, the transcriptional control of the nitrate assimilatory system is carried out by the synergistic action of the nitrate-specific transcription factor NirA and the major nitrogen-responsive regulator AreA. In this study, we identified the main components of the nitrate assimilation system in *F. fujikuroi* and studied the role of each of them regarding the regulation of the remaining components. We analyzed mutants with deletions of the nitrate-specific activator NirA, the nitrate reductase (NR), the nitrite reductase (NiR) and the nitrate transporter NrtA. We show that NirA controls the transcription of the nitrate assimilatory genes *NIAD*, *NIIA*, and *NRTA* in the presence of nitrate, and that the global nitrogen regulator AreA is obligatory for expression of most, but not all NirA target genes (*NIAD*). By transforming a NirA-GFP fusion construct into the Δ*NIAD*, Δ*NRTA*, and Δ*AREA* mutant backgrounds we revealed that NirA was dispersed in the cytosol when grown in the presence of glutamine, but rapidly sorted to the nucleus when nitrate was added. Interestingly, the rapid and nitrate-induced nuclear translocation of NirA was observed also in the Δ*AREA* and Δ*NRTA* mutants, but not in Δ*NIAD*, suggesting that the fungus is able to directly sense nitrate in an AreA- and NrtA-independent, but NR-dependent manner.

## Introduction

The phytopathogenic ascomycete fungus *Fusarium fujikuroi* produces a broad spectrum of interesting secondary metabolites (SM) including the phytohormones gibberellins (GA) and various pigments and mycotoxins. Biosynthesis and gene expression of 30 out of 47 SM gene clusters depend on availability of nitrogen (Wiemann et al., 2013; Tudzynski, 2014). Recently, we have shown that the global nitrogen regulators AreA and AreB play major roles in regulating nitrogen-controlled SM clusters (Michielse et al., 2014). Addition of preferred nitrogen sources, such as glutamine, to cultures led to rapid down-regulation of several nitrogen-repressed SM genes, e.g., the GA biosynthetic genes. By contrast, cultures supplied with nitrate showed a significant delay in repressing these clusters, probably due to the time-consuming conversion of nitrate to glutamine (Wagner et al., 2013). Although the ammonium permease (MepB) and the glutamine synthetase (GS) were shown to be involved in sensing ammonium and glutamine, respectively, nothing is known about potential nitrate sensors. Therefore, we wished to determine the molecular mechanisms involved in nitrate sensing, uptake, and gene regulation networks in *F. fujikuroi*.

The majority of fungi, with the exception of some yeast species, are able to assimilate nitrate or nitrite, the most abundant nitrogen ions present in soils and plants (Siverio, 2002; Song et al., 2007; Gorfer et al., 2011; Schinko et al., 2013). Utilization of nitrate requires its reduction to nitrite by the activity of nitrate reductase (NR) and reduction of nitrite to ammonium by nitrite reductase (NiR), respectively (Cove, 1979; Campbell and Kinghorn, 1990). This is an energy-consuming process and therefore, nitrate and nitrite are considered as unfavorable nitrogen sources that are only used when the preferred sources glutamine, glutamate, or ammonium are not available (Cove, 1979; Premakumar et al., 1979; Scazzocchio and Arst, 1989; Campbell and Kinghorn, 1990; Crawford and Arst, 1993; Siverio, 2002). It appears, therefore, that the nitrate assimilation system may be tightly regulated, as shown in many fungal species and plants. In *Aspergillus nidulans*, where the nitrate assimilation system has been thoroughly studied, the nitrate assimilation genes coding for NR (*NIAD*), NiR (*NIIA*), a nitrate/nitrite transporter (*NRTA*) and a specific nitrite transporter (*NITA*) are clustered, whilst a fifth gene, coding for a second transporter (*NRTB*) is located separately on the same chromosome (Cove, 1979; Brownlee and Arst, 1983; Johnstone et al., 1990; Unkles et al., 1991, 2001). These genes are only expressed when two conditions are met: low intracellular concentrations of preferred nitrogen sources (nitrogen-starvation conditions) and the simultaneous presence of nitrate or nitrite, which act as transcriptional inducers (reviewed by Marzluf, 1997). This expression pattern is subject to the synergistic action of the nitrate-specific regulator NirA (NIT4 in *Neurospora crassa*) and the global, nitrogen-dependent regulator AreA (NIT2 in *N. crassa*). In *A. nidulans*, the GAL4-type binuclea*r* Zn_2_Cys_6_ transcription factor (TF) NirA binds to non-palindromic consensus sequences (5′CTCCGHGG3′) in the bidirectional promotor of *NIAD* and *NIIA*, mediating their transcription (Burger et al., 1991; Punt et al., 1995; Strauss et al., 1998). While the *NIRA* gene itself is constitutively expressed irrespective of the nitrogen conditions (Burger et al., 1991), the NirA protein is subject to a strict post-translational regulation by cellular translocation. The addition of nitrate, nitrite, or chlorate leads to a rapid nuclear accumulation of NirA, even in the simultaneous presence of preferred nitrogen sources such as ammonium (Berger et al., 2006). This nitrate-responsive nuclear shuttling is subject to the interaction of NirA with the nuclear export factor KapK. Recently, it was shown that nitrate leads to a conformational change of NirA, based on the redox-status of methionine 169 in the nuclear export sequence (NES) of the protein. Nitrate-induced reduction of Met169 presumably masks the NES, thus leading to a disrupted NirA–KapK interaction and nuclear retention of the protein (Bernreiter et al., 2007; Gallmetzer et al., 2015). A similar nitrate-mediated nuclear shuttling was reported for the NirA-homolog NLP7 in the plant *Arabidopsis thaliana* (Marchive et al., 2013).

AreA, the second TF involved in control of the nitrate assimilation genes, belongs to the GATA family of Cys_2_-Cys_2_ TFs. In ascomycetes, it is considered as the major activator of genes involved in the utilization of alternative nitrogen sources, when preferred ones are absent. This kind of regulation, known as nitrogen metabolite repression (NMR), regulates not only components of the nitrate assimilation system, but also permeases and enzymes involved in the uptake and utilization of other nitrogen sources such as amino acids, GABA, or ammonium (Arst and Cove, 1973; Wiame et al., 1985; Marzluf, 1997; Magasanik and Kaiser, 2002; Macios et al., 2012; Michielse et al., 2014; Tudzynski, 2014; Pfannmüller et al., 2015). AreA itself is greatly repressed by high concentrations of nitrogen at the transcriptional, post-transcriptional, and post-translational levels (Kudla et al., 1990; Platt et al., 1996; Tao and Marzluf, 1999; Morozov et al., 2000, 2001; Caddick et al., 2006).

In *A. nidulans* and *N. crassa* it was shown that AreA/NIT2 is essential for *in vivo* DNA-binding of NirA/NIT4 to promoters of nitrate assimilation genes and for their expression. AreA/NIT2 binds to the bi-directional *NIIA*-*NIAD* promoter *in vivo* under nitrogen-limiting conditions and at the same time, mediates promoter binding of NirA/NIT4 by direct protein–protein interaction (Feng and Marzluf, 1998; Narendja et al., 2002; Muro-Pastor et al., 2004; Berger et al., 2006). Furthermore, AreA indirectly affects nuclear translocation of NirA due to the AreA-dependent expression of nitrate transporters, which are needed for uptake of extracellular nitrate (Berger et al., 2006).

In addition to AreA, a second GATA-TF involved in nitrogen-dependent gene regulation has been described in fungi, known as AreB in *Aspergillus* and *Fusarium* species. While AreB was initially described as a negative counterpart of AreA that acts as a major repressor of AreA-activated nitrogen catabolism genes (Wong et al., 2009), recent studies have shown that its function is more complex (Haas et al., 1997; Dzikowska et al., 2003). Recently, we have shown that AreA and AreB can act as synergistic activators or repressors of their shared target genes, which includes the genes of the GA biosynthesis cluster (Michielse et al., 2014). No studies have been made of any possible impact of AreB on nitrate assimilation.

The transcriptional activation of the *A. nidulans* nitrate assimilation gene cluster is also controlled by chromatin remodeling. Under non-inducing conditions, six nucleosomes are positioned at the bidirectional *NIIA*-*NIAD* promotor. In the presence of nitrate, however, all nucleosomes lose positioning, giving way to an open chromatin structure that enables gene transcription. This process depends on AreA-activity and was the first example of a GATA factor that is involved in chromatin remodeling (Muro-Pastor et al., 1999; Berger et al., 2006, 2008). Under nitrogen starvation conditions, AreA binds to GATA sites in the promoter region, mediating histone H3 acetylation by possible recruitment of histone acetyltransferases (Todd et al., 2005; Berger et al., 2008). Additionally, the nitrate-induced TF NirA also influences the chromatin status, but only under nitrate induction conditions in an H3 acetylation-independent manner (Berger et al., 2008). AreA-dependent chromatin remodeling at the genomic locus of nitrate assimilation genes was also reported in *Fusarium oxysporum* (Malardier et al., 1989; López-Berges et al., 2010, 2014).

In this work, we studied the system of nitrate assimilation in *F. fujikuroi*, including its uptake, incorporation into metabolism and a possible direct sensing of nitrate and nitrite. We identified the main functional components involved in nitrate uptake and assimilation, including the genes encoding the NR, NiR, the main nitrate transporter NrtA and the TF NirA and studied their expression in response to the presence of nitrate, nitrite, and glutamine. We also analyzed the regulatory roles of the pathway-specific TF NirA and the global nitrogen-regulators AreA and AreB. Apart from transcriptional regulation, we investigated the regulation of the subcellular localization of the NirA::GFP fusion protein in the wild-type (Wt) and deletion mutants in response to different nitrogen sources.

## Results

### Identification of *F. fujikuroi* Nitrate Assimilation Genes

To identify the respective orthologous nitrate assimilation genes, we performed a BLAST search on the *F. fujikuroi* genome database (Wiemann et al., 2013) with protein sequences of the NiR, the nitrate transporter NrtA and the TF NirA of *A. nidulans* and *F. oxysporum* as queries. The *F. fujikuroi NIAD* gene, coding for a putative NR, was previously identified (Tudzynski et al., 1996). For each sequence, one putative homolog with high sequence similarity was identified (**Table 1**) and the respective *F. fujikuroi* genes will be referred to as *NIAD* (*FFUJ_12277*, encoding NR), *NIIA* (*FFUJ_06099*, encoding NiR), *NRTA* (FFUJ_00934), and *NIRA* (FFFUJ_04567), respectively.

**Table 1:** Genome position of *F. fujikuroi* genes coding for putative components of the nitrate assimilation system and homologies to other fungal species.

		*Fusarium fujikuroi*					Homology					
		Binding sites		Genome position			*Aspergillus nidulans*			*Fusarium oxysporum*		
Name	Function	NirA	GATA	Gene ID	Contig	Position	Gene ID	Score	*E*-value	Gene ID	Score	*E*-value
***NIAD***	Nitrate reductase	1	9	FFUJ_ 12277	FFUJ_ chr08	1520527-1517747	AN1006	969	0	FOXG_04181	1771	0
***NIIA***	Nitrite reductase	2	9	FFUJ_ 06099	FFUJ_ chr06	2445443-2442018	AN1007	1293	0	FOXG_03192	2237	0
***NRTA***	Nitrate transporter	1	7	FFUJ_ 00934	FFUJ_ chr01	3766795-3768471	AN1008	460	6.90 E-130	FOXG_00635	971	0
***NIRA***	Gal4-type Zn2-C6 TF	0	3	FFUJ_ 04567	FFUJ_ chr02	2390471-2393213	AN0098	754	0	FOXG_06396	1709	0

Notably, every single gene encoding one of the putative nitrate assimilation components is located on a different chromosome in *F. fujikuroi* (**Table 1**). This genomic arrangement is different from the one in *A. nidulans*, where *NIAD*, *NIIA*, and *NRTA* are located on the same chromosome in close proximity forming a co-regulated gene cluster (Cove, 1979; Brownlee and Arst, 1983; Johnstone et al., 1990). The scattering of the nitrate assimilation genes in the genome of *F. fujikuroi* may indicate a different, more uncoupled transcriptional regulation compared to *A. nidulans*.

The promotor sequences upstream of the *NIRA*, *NIAD*, *NIIA*, and *NRTA* genes were searched for putative binding sites of NirA (non-palindromic recognition sequence 5′CTCCGHGG3′) and GATA-TFs like AreA (double GATA/TATC sequence elements with a distance of <30 bp). The promoter sequences of *NIAD*, *NIIA*, and *NRTA* were found to contain at least one putative NirA-binding motif, while multiple double GATA-binding sites were present in the 5′ non-coding regions of all four genes (**Table 1**).

### NR, NiR, NrtA, and NirA Are the Main Components of Nitrate Assimilation in *F. fujikuroi*

**FIGURE 1:**
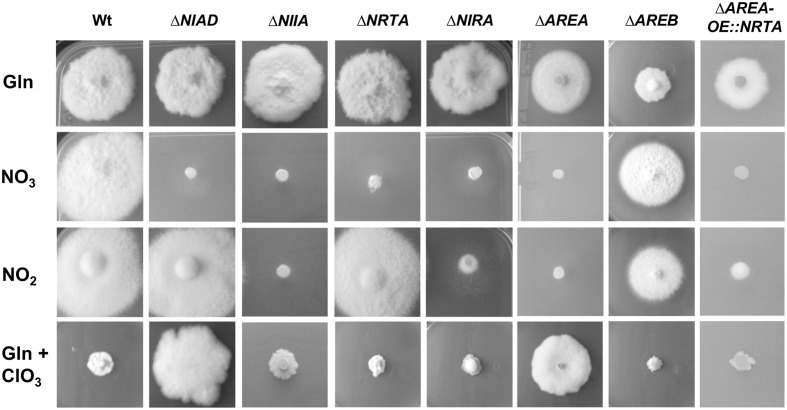
**Growth analysis of the *F. fujikuroi* Wt and the Δ*NIAD*, Δ*NIIA*, Δ*NRTA*, Δ*NIRA*, Δ*AREA*, Δ*AREB*, Δ*AREA-OE::NRTA* mutants on various nitrogen sources**. The strains were cultivated on solidified synthetic ICI medium supplemented with 6 mM glutamine (Gln), 12 mM sodium nitrate (NO_3_), 12 mM sodium nitrite (NO_2_), or 6 mM glutamine with 30 mM potassium chlorate (Gln + ClO_3_) at 28°C for 4 days.

To elucidate the functions of the putative *F. fujikuroi* nitrate assimilatory genes, deletion mutants of *NIIA*, *NRTA*, and *NIRA* were generated. The *F. fujikuroi* Wt, the newly generated knockout strains and the already available Δ*NIAD* (Tudzynski et al., 1996), Δ*AREA* (Tudzynski et al., 1999), and Δ*AREB* (Michielse et al., 2014) deletion mutants were incubated for 4 days on solidified synthetic ICI minimal medium, supplemented with different nitrogen sources (**Figure 1**). All mutant strains were able to grow in a Wt-like manner on glutamine, with the exception of Δ*AREB*, which showed a slightly reduced growth. In contrast, only the Wt and Δ*AREB* were able to grow with nitrate, clearly demonstrating that NR, NiR, NrtA, NirA, and AreA are crucial components for the use of nitrate as a nitrogen source. This was also reflected by a drastically reduced biomass formation of these mutants when cultivated in liquid cultures with nitrate as the sole nitrogen source (Supplementary Figure S1A).

On solid medium with nitrite, the colony diameter of the Wt was similar to that on glutamine and nitrate. However, the aerial mycelium appeared to be thinner, suggesting less biomass formation (**Figure 1**). This was confirmed by cultivation of the Wt in liquid medium with glutamine, nitrate or different concentrations of nitrite (Supplementary Figure S1B) and is probably caused by toxic effects of nitrite that inhibit growth of the fungus (Wodzinski et al., 1978; Crawford and Arst, 1993; Cabrera et al., 2014). The Δ*NIAD* and Δ*NRTA* mutants showed Wt-like growth on nitrite, whereas the Δ*AREB* mutant grew with a slightly reduced colony diameter compared to the Wt (**Figure 1**). In contrast, no growth on nitrite medium has been observed for the Δ*NIIA*, Δ*NIRA*, and Δ*AREA* mutants, demonstrating that NiR is the only nitrite-reducing enzyme, and that the TFs NirA and AreA, but not AreB, are essential for the assimilation of nitrite (**Figure 1**). Furthermore, the Wt-like growth of Δ*NRTA* on nitrite showed that this transporter is essential for transport of nitrate, but not of nitrite (**Figure 1**).

We also studied the sensitivity of all strains to chlorate (ClO_3_), a structural analog of nitrate. Chlorate is imported into the cell by nitrate transporters and acts as a substrate of the NR which reduces it to the highly reactive and toxic chlorite (ClO_2_), thereby killing the cells (Cove, 1976; Brownlee and Arst, 1983; van Wijk et al., 1998). Accordingly, the *F. fujikuroi* Wt showed almost no growth on medium supplemented with chlorate due to the activity of the NR. The same was the case for the Δ*NIIA*, Δ*NRTA*, Δ*NIRA*, and Δ*AREB* mutants indicating that they are able to reduce chlorate to chlorite. However, deletion of *NIAD* and *AREA* mediated resistance to chlorate, probably due to the loss or drastic down-regulation of NR activity, respectively, in these mutants (**Figure 1**).

To elucidate the impact of AreA on the nitrate assimilation in more detail, we constitutively expressed the nitrate transporter gene *NRTA* in the background of the Δ*AREA* deletion mutant. The resulting strain Δ*AREA-OE::NRTA* was unable to grow on nitrate and nitrite medium, but in contrast to the Δ*AREA* mutant, was sensitive to chlorate due to the AreA-independent import of nitrate by the activity of the transporter NrtA and most likely low but sufficient activity of the NR in the Δ*AREA* background.

Taken together, our growth analysis showed that NR, NiR, and NrtA are crucial components of the *F. fujikuroi* nitrate assimilation system as they are all needed for growth on nitrate, whereas NiR is essential for growth on nitrite. NrtA seems to be the major nitrate transporter of *F. fujikuroi*, but it is not or not solely involved in the uptake of nitrite. In addition, our plate assays clearly show that both the TF NirA and the global GATA-TF AreA are involved in the regulation of the nitrate and nitrite assimilation system.

### Is Nitrate Directly Sensed and Causes NMR?

In *F. fujikuroi*, it was previously shown that the expression of many SM gene clusters is subject to NMR in an AreA-dependent (e.g., GA biosynthesis genes) or AreA-independent [e.g., bikaverin (BIK) biosynthesis genes] manner (Candau et al., 1992; Mihlan et al., 2003; Wiemann et al., 2009, 2013; Tudzynski, 2014). The main effector mediating rapid and strong repression of these two SM clusters was shown to be glutamine, while addition of nitrate to cultures led to a significantly delayed down-regulation of these genes compared to glutamine (Wagner et al., 2013).

**FIGURE 2:**
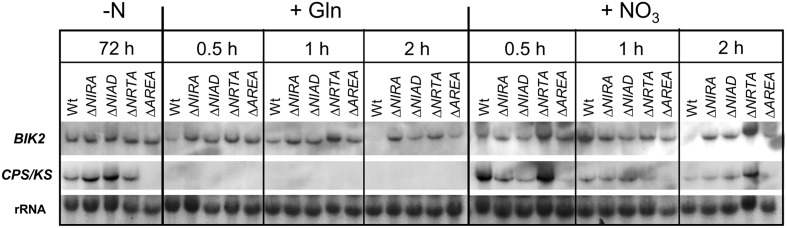
**Time-course of GA and BIK biosynthetic gene expression in response to high concentrations of glutamine and nitrate**. The *F. fujikuroi* Wt and the Δ*NIRA*, Δ*NIAD*, Δ*NRTA*, Δ*AREA* mutants were cultivated in liquid synthetic ICI medium at 28°C for 3 days (-N). Cultures were then supplemented with 60 mM glutamine (+Gln) or 60 mM sodium nitrate (+NO_3_). Mycelia were harvested before (-N) or 0.5, 1, or 2 h after the addition of the respective nitrogen sources and expression of *CPS/KS* (copalyl diphosphate/kaurene-synthase, GA-cluster) and *BIK2* (monooxygenase, BIK-cluster) was analyzed by Northern blot.

However, it is difficult to distinguish between a direct (though delayed) regulatory effect of nitrate itself and an indirect effect via its conversion to glutamine. Therefore, we investigated the different effects of nitrate and glutamine on the expression of GA and BIK genes during a time course of 2 h in the Wt and the Δ*NIAD*, Δ*NRTA*, Δ*NIRA*, and Δ*AREA* mutants. By using these mutants for expression analyses we were able to further differentiate between nitrate- and glutamine-mediated effects, because the uptake and/or the metabolization of nitrate and nitrite was clearly abolished in these mutants (**Figure 1**). Since the deletion mutants are unable to grow with nitrate as the sole nitrogen source, we cultivated the *F. fujikuroi* Wt and the Δ*NIAD*, Δ*NRTA*, Δ*NIRA*, and Δ*AREA* mutants initially in synthetic medium with 6 mM glutamine for 3 days. At this time (72 h), glutamine was depleted and the fungus coped with nitrogen-starvation (-N) as indicated by high expression of GA and BIK biosynthetic genes (**Figure 2**). The cultures were then supplemented with either 60 mM glutamine or 60 mM nitrate, and mycelia were harvested 0.5, 1, or 2 h after the addition of nitrogen to study the expression of the GA and BIK biosynthetic genes, *CPS/KS* and *BIK2*, respectively, by Northern blot analysis (**Figure 2**).

Under nitrogen-starvation (after 72 h growth in 6 mM glutamine), *CPS/KS* and *BIK2* were highly expressed in the Wt and all deletion mutants, except for *CPS/KS* in the Δ*AREA* mutant due to the AreA-dependent expression of GA genes (Mihlan et al., 2003). The addition of high concentrations of glutamine resulted in rapid and total loss of *CPS/KS* expression in all strains, while *BIK2* expression was detected in the Wt up to 1 h after glutamine addition. Surprisingly, a de-regulation of *BIK* expression was observed in Δ*NIRA*, Δ*NIAD*, and Δ*NRTA* after addition of glutamine, because expression was clearly detected in these mutants up to 2 h in contrast to the Wt.

When high concentrations of nitrate were added, transcripts of both *CPS/KS* and *BIK* were clearly visible in the Wt up to 2 h after nitrate addition, though with decreasing intensity (**Figure 2**). This time-delay of gene repression in response to nitrate could be an indication that nitrate is not directly sensed as an effector of NMR, and that down-regulation of genes began only after its conversion into glutamine, the real effector of NMR. This hypothesis is further supported by the fact that no obvious repression was observed up to 2 h after nitrate-addition in Δ*NIRA*, Δ*NIAD*, and Δ*NRTA* mutants. These mutants were unable to utilize nitrate, and therefore no accumulation of the main NMR-effector glutamine could take place.

In summary, our expression analysis revealed that the genes of the GA and BIK cluster are quickly repressed by glutamine, but are not directly affected by nitrate. Instead, the delayed repression in comparison to that for glutamine is due to the time-consuming conversion of nitrate to glutamine. In contrast to the Wt, we did not observe a significant repression of GA and BIK genes in the Δ*NIRA*, Δ*NIAD*, and Δ*NRTA* mutants in response to nitrate as all these mutants are unable to metabolize nitrate. The data indicated that nitrate is not sensed directly as an effector of NMR in *F. fujikuroi*.

### Transcriptional Regulation of Nitrate Assimilatory Genes

**FIGURE 3:**
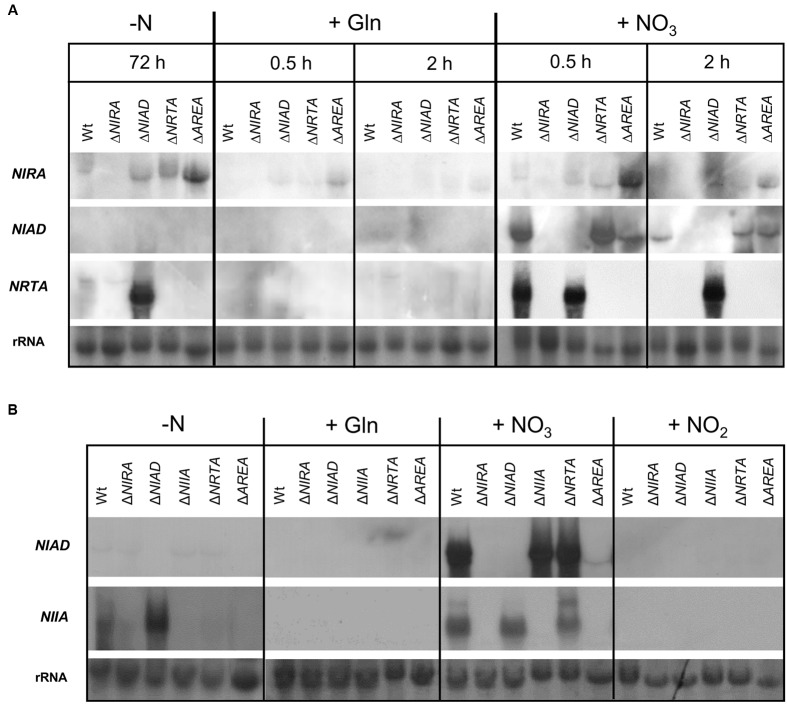
**Expression of nitrate assimilation genes in response to different nitrogen sources**. The *F. fujikuroi* Wt and the Δ*NIRA*, Δ*NIAD*, Δ*NRTA*, Δ*AREA* mutants were cultivated in liquid synthetic ICI medium at 28°C for 3 days (-N). **(A)** Cultures were supplemented with 60 mM glutamine (+Gln) or 60 mM sodium nitrate (+NO_3_). Mycelia were harvested before (-N) or 0.5 h or 2 h after the addition of the respective nitrogen sources and expression of *NIRA*, *NIAD*, and *NRTA* was analyzed by Northern blot. **(B)** Cultures were supplemented with 30 mM glutamine (+Gln), 12 mM sodium nitrate (+NO_3_) or 12 mM sodium nitrite (+NO_2_). Mycelia were harvested before (-N) or 0.5 h after the addition of the respective nitrogen sources and expression of *NIAD* and *NIIA* was analyzed by Northern blot.

In *A. nidulans*, the expression of the co-regulated nitrate assimilation genes *NIAD*, *NIIA*, and *NRTA* is induced by nitrate and nitrite and depends on the activity of both the specific TF NirA and the global, nitrogen-dependent regulator AreA (Berger et al., 2006). To study the transcriptional regulation of these genes in *F. fujikuroi*, the Wt and the Δ*NIAD*, Δ*NRTA*, Δ*NIRA*, and Δ*AREA* mutants were first cultivated for 3 days with 6 mM glutamine (nitrogen-limiting conditions) before high concentrations of either 60 mM glutamine or 60 mM nitrate were added to the cultures. Expression of the genes *NIRA*, *NIAD*, and *NRTA* was studied before, and 0.5 or 2 h after the addition of nitrogen (**Figure 3A**).

A weak or no expression of the TF-encoding gene *NIRA* was detected in the Wt under nitrogen-starvation conditions and after the addition of glutamine, respectively, indicating that *NIRA* is subject to NMR. After addition of nitrate, *NIRA* transcripts were detected at 0.5 h, but were completely absent after 2 h, likely due to the accumulation of glutamine similar to the delayed nitrate-repression of the GA and BIK cluster genes (**Figure 2**). Accordingly, NirA expression was still detected 2 h after nitrate addition in the Δ*NIAD* and Δ*AREA* mutants, which are unable to convert nitrate to glutamine (**Figure 3A**). Surprisingly, the expression levels of *NIRA* were significantly elevated in Δ*AREA* compared to the Wt under all tested conditions, indicating a repressive impact of AreA on *NIRA* expression.

Expression of *NIAD* and *NRTA* was not detected in any strain under nitrogen-starvation conditions or after addition of glutamine (**Figure 3A**). However, 0.5 h after addition of nitrate, strong expression of both genes was detected in the Wt, but was significantly reduced or abolished at 2 h. It was notable that the same nitrate-dependent induction of *NIAD*-expression was detected in the Δ*NRTA* mutant – which, based on our plate assays, was hitherto assumed to be unable to import nitrate into the cell (**Figure 1**). Furthermore, a strong expression of *NRTA* was observed in the Δ*NIAD* mutant under nitrogen-limiting conditions without nitrate. However, the addition of glutamine immediately resulted in *NRTA*-repression. After addition of nitrate, the expression was still high at 2 h when the Wt no longer showed *NRTA* expression, probably due to the unmetabolized presence of nitrate in this mutant (**Figure 3A**).

In a second experiment, we compared the effects of nitrate and nitrite on the expression of *NIAD* and *NIIA*. Therefore, the *F. fujikuroi* Wt, the Δ*NIAD*, Δ*NIIA*, Δ*NRTA*, Δ*NIRA*, and Δ*AREA* mutants were cultivated for 3 days as described above and then supplemented with 60 mM glutamine, 12 mM nitrate, or 12 mM nitrite for 0.5 h each (**Figure 3B**). *NIAD* and *NIIA* expression followed the same nitrate-induced pattern as in the previous experiment (**Figures 3A,B**). In contrast, no expression of *NIAD* or *NIIA* was observed upon the addition of nitrite, the substrate of NiR (**Figure 3B**). In a similar manner to *NRTA, NIIA* was strongly expressed in the Δ*NIAD* mutant under nitrogen-starvation conditions without the addition of nitrate (**Figure 3B**).

The expression pattern of the nitrate assimilation genes in the background of the Δ*NIRA* and Δ*AREA* deletion mutants provide insights to the possible regulatory role of the two TFs (**Figure 3**). In Δ*NIRA*, no expression was observed for *NIAD*, *NIIA*, and *NRTA* under all tested conditions, clearly showing that NirA acts as an obligate and specific transcriptional activator for these genes. Similarly, no expression of *NIIA* and *NRTA* was observed in Δ*AREA*. A weak, but clearly visible, expression of *NIAD* was detected in Δ*AREA* mutant after addition of nitrate indicating that AreA probably acts as a strong, though not an obligate transcriptional activator of *NIAD*.

Taken together, our expression analysis revealed that the main nitrate assimilation genes *NIAD*, *NIIA*, and *NRTA* are repressed by high concentrations of glutamine and highly expressed upon induction with nitrate, but not nitrite. The TFs NirA and AreA are both needed for transcriptional activation of *NIIA* and *NRTA*, and NirA for transcription of *NIAD*. AreA acts also as a strong positive regulator of *NIAD*, but is not essential for its expression.

### NirA Is Regulated by Nucleocytoplasmatic Shuttling

Apart from a nitrate-dependent regulation at the transcriptional level, the pathway-specific TF NirA was shown to be regulated on a post-translational level in *A. nidulans*, by a nitrate-dependent shuttling from a primary cytosolic localization to an accumulation inside the nucleus (Berger et al., 2006). To investigate a similar localization-dependent regulation of NirA in *F. fujikuroi*, a NirA::GFP fusion construct was constitutively expressed in the Δ*NIRA* mutant, resulting in strain Δ*NIRA-NIRA::GFP*. A plate assay showed that the fusion construct was functional because the growth defect shown by Δ*NIRA* was fully restored on nitrate and nitrite medium by expression of NirA::GFP (Supplementary Figure S2).

**FIGURE 4:**
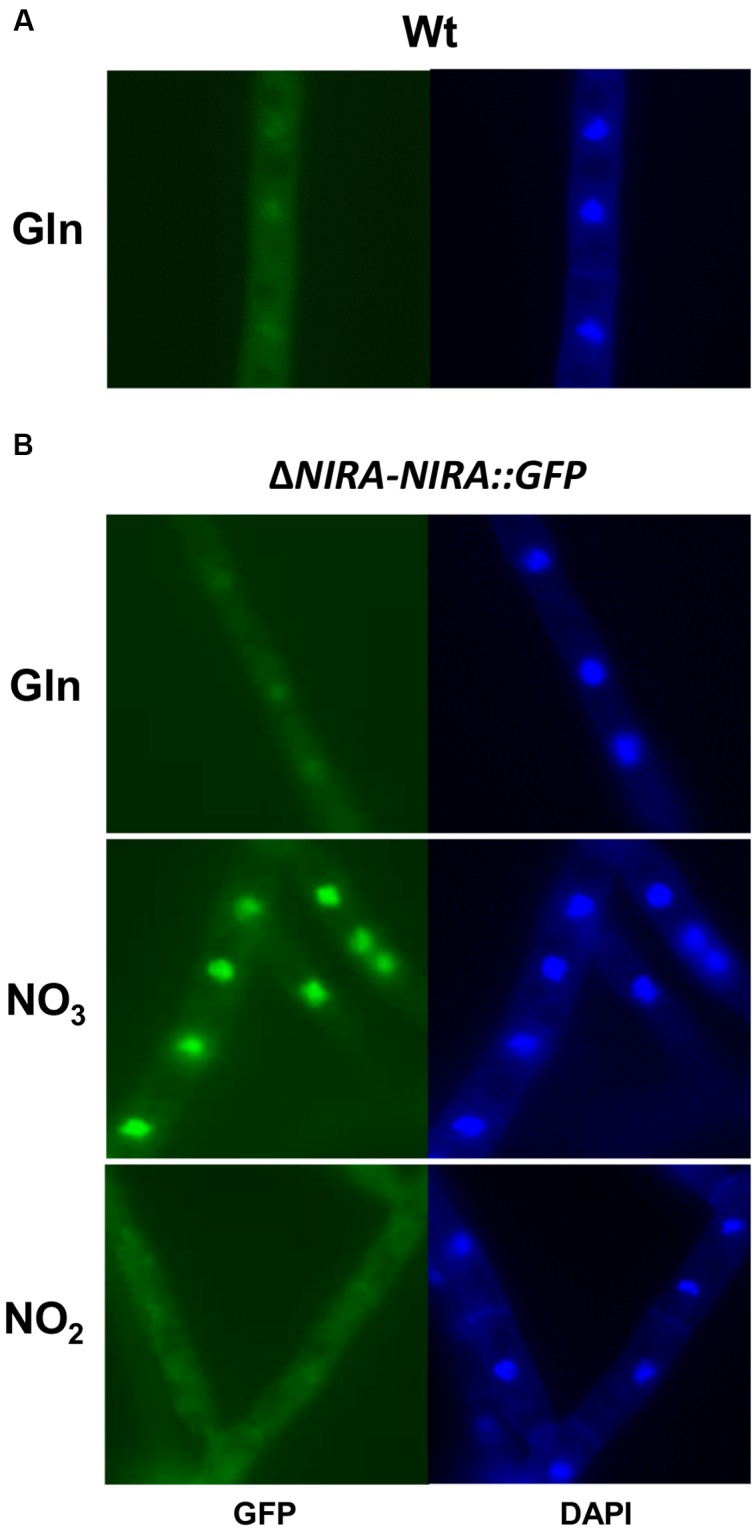
**Nitrate-dependent nuclear translocation of the NirA::GFP fusion protein**. The *F. fujikuroi* Wt **(A)** and the Δ*NIRA* mutant transformed with a constitutively expressed NirA::GFP fusion construct **(B)** was cultivated in liquid synthetic ICI medium with 6 mM glutamine for 2 days at 28°C. Cells were observed by fluorescence microscopy (GFP) less than 1 min after the addition of 6 mM glutamine (Gln), 12 mM sodium nitrate (NO_3_), or 12 mM sodium nitrite (NO_2_). Additionally, cells were stained with Hoechst solution for visualization of nuclei (DAPI).

For microscopic observation, the Δ*NIRA-NIRA::GFP* strain and the Wt (as a control) were cultivated in liquid ICI medium with 6 mM glutamine until the cells were starved for nitrogen. The Δ*NIRA-NIRA::GFP* cultures were then supplemented with either 6 mM glutamine, 12 mM nitrate, or 12 mM nitrite, and the cellular localization of NirA::GFP was examined immediately after (<30 s) by fluorescence microscopy. Cells were simultaneously stained with Hoechst solution for visualization of nuclei (**Figure 4**). With the glutamine supplement, the Δ*NIRA-NIRA::GFP* cells showed a very faint fluorescence inside round structures, overlapping with the Hoechst staining of nuclei (**Figure 4B**). However, the same faint nuclear signals were detected in the Wt, indicating that it is not a specific GFP signal, but instead a non-specific background fluorescence caused by the Hoechst solution as seen in the GFP filter set (**Figure 4A**). Additionally, we never observed a similar nuclear GFP signal with glutamine without simultaneous Hoechst staining. Therefore, the NirA::GFP fusion protein does not appear to be localized to particular structures under glutamine conditions and is most likely distributed evenly inside the cell. Similarly, no specific localization of the fusion protein was observed after the addition of nitrite (**Figure 4B**). In contrast, a strong fluorescence signal was detected inside the nucleus of the Δ*NIRA-NIRA::GFP* strain immediately after addition of nitrate (**Figure 4B**).

**FIGURE 5:**
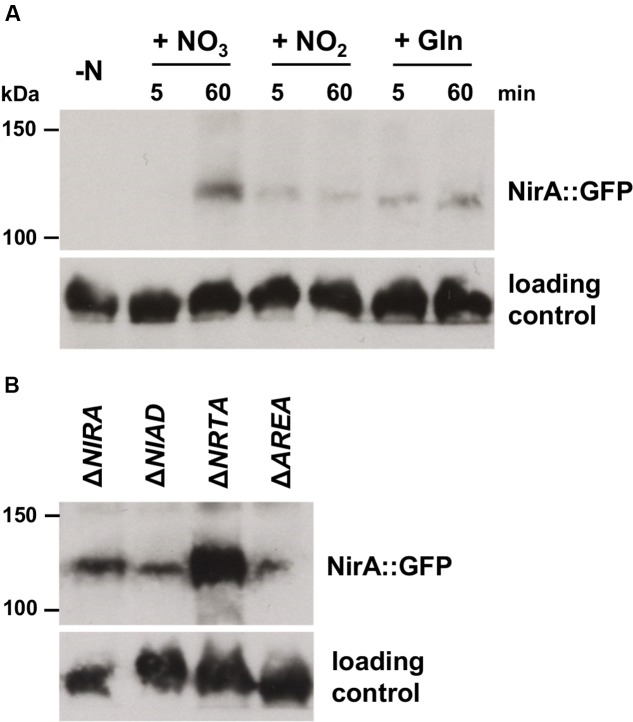
**Abundance of NirA::GFP protein after exposure to different nitrogen sources. (A)** The *F. fujikuroi* Δ*NIRA* mutant transformed with a constitutively expressed NirA::GFP fusion construct was cultivated in liquid synthetic ICI medium with 6 mM glutamine for 3 days at 28°C. Cultures were harvested before (-N) and 5 min as well as 60 min after addition of 12 mM sodium nitrate (+ NO_3_), 12 mM sodium nitrite (+ NO_2_) and 60 mM glutamine (+ Gln). **(B)** The *F. fujikuroi* Δ*NIRA*, Δ*NIAD*, Δ*NRTA*, and Δ*AREA* mutants transformed with a constitutively expressed NirA::GFP fusion construct was cultivated in liquid synthetic ICI medium with 6 mM glutamine for 3 days at 28°C. Cultures were harvested 60 min after addition of 12 mM sodium nitrate. Total protein was extracted and analyzed by Western blot using polyclonal anti-GFP antibodies for detection of NirA::GFP. Hybridization with anti-β-actin antibodies was used as protein loading control. Molecular weights are indicated in kDa.

To assess the total protein level of NirA::GFP under different nitrogen conditions, we performed an immunoblot analysis. Nitrogen-starved cultures of Δ*NIRA-NIRA::GFP* were supplemented with 12 mM nitrate, 12 mM nitrite, or 60 mM glutamine. Cells were harvested before the addition of nitrogen, and 5 and 60 min after addition of nitrogen, and the total protein was extracted for Western blot analysis (**Figure 5A**). The fusion protein was neither detected before, nor 5 min after addition of nitrate, and only a faint signal was visible 5 and 60 min after addition of nitrite or glutamine. In contrast, a strong signal of NirA::GFP was detected 60 min after addition of nitrate demonstrating that only nitrate, but not nitrite or glutamine, causes a significant accumulation of the NirA protein (**Figure 5A**). It should be noted that this accumulation of the NirA::GFP protein took between 5 and 60 min, whereas the strong nuclear accumulation was completed significantly faster in a time-span of less than 30 s (**Figure 4A**).

**FIGURE 6:**
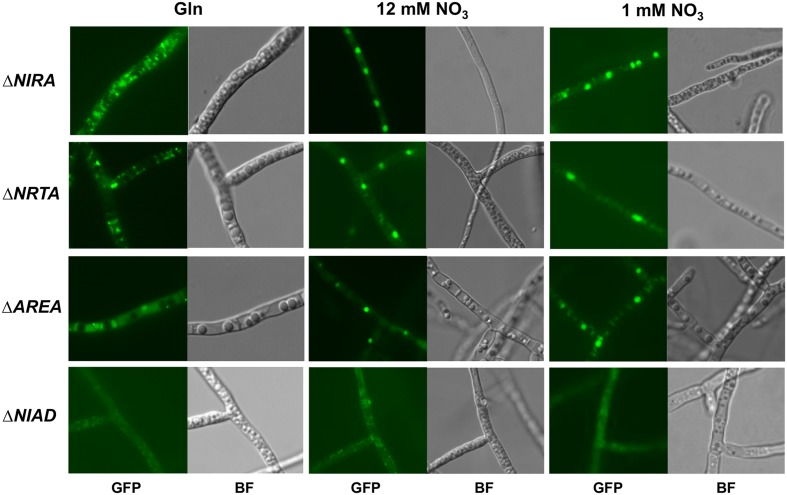
**Involvement of NrtA, AreA, and NiaD in nitrate-dependent nuclear translocation of the NirA::GFP fusion protein**. The *F. fujikuroi* Δ*NIRA*, Δ*NRTA*, Δ*AREA*, and Δ*NIAD* mutants transformed with a constitutively expressed NirA::GFP fusion construct were cultivated in liquid synthetic ICI medium with 6 mM glutamine for 2 days at 28°C. Cells were observed by fluorescence (GFP) and brightfield (BF) microscopy after the addition of 6 mM glutamine (Gln), 12 mM sodium nitrate (12 mM NO_3_), or 1 mM sodium nitrate (1 mM NO_3_).

In order to investigate a possible impact of the other components of the nitrate assimilation system on NirA-localization, we also expressed the NirA::GFP fusion protein in the background of the Δ*NRTA*, Δ*AREA*, and Δ*NIAD* deletion mutants. The resulting strains Δ*NRTA-NIRA::GFP*, Δ*AREA-NIRA::GFP*, and Δ*NIAD-NIRA::GFP*, as well as strain Δ*NIRA-NIRA::GFP* (control) were cultivated as described above, after which 6 mM glutamine or two different concentrations of nitrate (12 mM or 1 mM) were added. The cells were then examined immediately afterward by fluorescence microscopy (**Figure 6**).

We observed a similar nitrate-induced nuclear accumulation of NirA::GFP as in our previous experiment (**Figure 4**), even with the lowest concentration of nitrate (1 mM) (**Figure 6**). The same localization pattern of NirA::GFP was also observed in the Δ*NRTA* and Δ*AREA* backgrounds suggesting that small amounts of nitrate are present in the cells. In contrast, no nuclear accumulation of NirA::GFP in response to nitrate was observed in the Δ*NIAD* background. We observed only a faint, evenly distributed GFP fluorescence in the cytosol, indicating that the NR appears to be involved in regulating the nitrate-dependent NirA-localization.

To address the possibility that the NirA::GFP protein is degraded in the Δ*NIAD* mutant, in contrast to the other mutants, the fusion protein was assessed 60 min after addition of nitrate in the different deletion backgrounds by Western blot analysis (**Figure 5B**). Since the fusion protein was ectopically expressed in all strains, the basal expression level might differ depending on the number of genomic integrations and the integration loci. Therefore, a direct quantitative comparison of the NirA::GFP-levels in Δ*NIRA*, Δ*NIAD*, Δ*NRTA*, and Δ*AREA* was not possible. However, the fusion protein was clearly detected in all four strains under nitrate conditions (**Figure 5B**), proving that the lack of nuclear NirA::GFP localization in Δ*NIAD* is the result of a disrupted nucleocytoplasmic shuttling and not an indirect effect caused by absence of the fusion protein.

Taken together, microscopic observations of the subcellular localization of the NirA::GFP fusion protein revealed that the TF is regulated on a post-translational level by nitrate- but not nitrite-dependent fast nuclear translocation and slower *de novo* NirA protein synthesis and potentially nitrate-induced protein stabilization. Evidence suggests that the NirA-translocation apparently depends on the NR, while the nitrate transporter NrtA and the GATA-TF AreA are not involved.

## Discussion

In this work, we studied the molecular mechanisms of the nitrate assimilation system in *F. fujikuroi*. We identified and functionally characterized the main genes involved in nitrate and nitrite assimilation and investigated the impact of the pathway-specific TF NirA and the global nitrogen regulator AreA on their expression.

### NR and NiR Are the Sole Nitrate and Nitrite Reductases, Respectively, in *F. fujikuroi*

Our sequence analysis revealed that the genes *NIAD* and *NIIA* are the only genes in the genome of *F. fujikuroi* that encode NR and NiR, respectively. Deletion of the two genes confirmed their putative functions: Δ*NIAD* was no longer able to use nitrate, while Δ*NIIA* was unable to use nitrate or nitrite. Additionally, deletion of *NIAD* mediated resistance to chlorate (**Figure 1**). The growth analysis also clearly showed that the loss of NR or NiR activity by deleting *NIAD or NIIA*, respectively, cannot be compensated by any other enzyme. This proves that *NIAD* encodes the sole functional NR and *NIIA* encodes the sole functional NiR of *F. fujikuroi*.

### Nitrate-Uptake Depends on the Transporter NrtA

In contrast to *A. nidulans*, where two functional nitrate transporters have been described and characterized (Brownlee and Arst, 1983; Unkles et al., 1991, 2001), we identified only a homolog of one of the *A. nidulans* nitrate transporters, NrtA. This is similar to *F. oxysporum*, where only a single nitrate transporter homolog is also present (López-Berges et al., 2014).

The generated *F. fujikuroi* Δ*NRTA* mutant was not able to grow on nitrate medium, clearly showing that NrtA is the major nitrate transporter (**Figure 1**). However, NrtA cannot be the only nitrate/chlorate transporter, because the deletion of *NRTA* did not mediate resistance to chlorate. In addition, nitrate-induced nuclear translocation of NirA::GFP in the Δ*NRTA* background suggested that at least small amounts of nitrate can be transported into the cell by alternative transporters. Furthermore, the Wt-like growth of Δ*NRTA* with nitrite as the sole nitrogen-source demonstrated that nitrite is internalized by other transporters. However, it cannot be excluded that NrtA is also able to transport nitrite.

A distinct transporter for uptake of nitrite named NitA/Nrt2, has already been described in *A. nidulans* (Wang et al., 2008) and *F. oxysporum* (López-Berges et al., 2014), respectively. A BLAST analysis of the *F. fujikuroi* genome revealed the presence of a putative nitrite transporter (FFUJ_03993) with significant homologies to NitA/Nrt2 (data not shown). Furthermore, the studies in *A. nidulans* showed that NitA is a specific transporter for the uptake of nitrite, while the two nitrate transporters NrtA and NrtB are able to additionally transport nitrite (Wang et al., 2008). A similar dual role as a nitrate and nitrite transporter may also be the case for NrtA in *F. fujikuroi* and should be investigated further in the course of characterizing the putative nitrite transporter.

### Expression of Nitrate Assimilation Genes Depends on Nitrate and the Regulator NirA, But Not Completely on AreA

As in other fungi (Cove, 1979; Dunn-Coleman et al., 1981; Crawford and Arst, 1993; Marzluf, 1997; Siverio, 2002), the expression of the nitrate assimilation genes *NIAD*, *NIIA*, and *NRTA* was induced by the addition of nitrate to the growth medium and repressed by high concentrations of glutamine (**Figure 3**). An induction of *NIIA* and *NRTA* gene expression was also observed in the *NIAD* deletion mutant without the addition of nitrate, most likely due to trace amounts of nitrate that accumulate in this strain over time. A similar effect on gene expression was also described for the *A. nidulans* Δ*NIAD* mutant and termed as ‘pseudo-constitutive’ expression (Schinko et al., 2013).

Additionally, the TF NirA apparently acts as an obligate transcriptional activator of all *F. fujikuroi* nitrate assimilation genes, as no expression of *NIAD*, *NIIA*, and *NRTA* was observed in the Δ*NIRA* mutant. We identified potential NirA-binding motifs in the promotor sequences of all three genes, suggesting that NirA most likely activates their expression by directly binding to the respective promoters. A similar transcriptional activation of nitrate assimilation genes by promoter-binding of NirA/Nit4 has been described in *A. nidulans* and *N. crassa* (Burger et al., 1991; Yuan et al., 1991; Punt et al., 1995; Feng and Marzluf, 1998; Strauss et al., 1998).

Accordingly, the *NIRA* mutant is unable to grow on nitrate or nitrite, indicating that the NrtA, NR, and NiR activities must be drastically reduced in this mutant. However, despite the obligate dependency of the nitrate assimilation on NirA, the Δ*NIRA* mutant was unable to grow on medium supplemented with the toxic nitrate analog chlorate, indicating that at least low NR activity must be retained. This low activity is most likely not sufficient to support the growth on nitrate. This becomes apparent when compared to the Δ*NIAD* mutant, which displayed full chlorate resistance due to total loss of NR activity (**Figure 1**).

In *A. nidulans* and *N. crassa* it has been shown that the GATA-TF AreA/Nit2 is essential for the promoter-binding of NirA/Nit4 which involves direct protein–protein interaction (Arst and Cove, 1973; Feng and Marzluf, 1998; Muro-Pastor et al., 1999; Narendja et al., 2002; Berger et al., 2006). Furthermore, it has been shown that AreA is involved in opening the chromatin structure at the genomic loci of the nitrate assimilation genes in *A. nidulans* and *F. oxysporum* (Muro-Pastor et al., 1999; Todd et al., 2005; Berger et al., 2008; López-Berges et al., 2010, 2014).

Our results indicated that AreA acts as a strong positive regulator of the nitrate assimilation genes in *F. fujikuroi*: the Δ*AREA* mutant was not able to grow on nitrate and nitrite, but in turn was resistant to chlorate (**Figure 1**). Consequently, no expression of *NRTA* or *NIIA* was observed upon deletion of *AREA*. Surprisingly, the expression of *NIAD* was only down-regulated in Δ*AREA*, but not totally abolished. As the *NIAD* transcription does not completely depend on AreA, the aforementioned chlorate resistance of the Δ*AREA* mutant is most likely caused by the completely blocked import of chlorate due to the down-regulation of *NRTA* and additional uncharacterized transporters with non-specific transport activity for nitrate/chlorate by the global TF AreA. The constitutive, AreA-independent, expression of the main nitrate/chlorate transporter NrtA in the Δ*AREA* background (**Figure 1**) did not restore growth on nitrate medium but restored the susceptibility to chlorate. These data clearly demonstrate that, in accordance with our expression analysis, a low NR activity must be left in Δ*AREA* which is insufficient for growth on nitrate but adequate for conversion of chlorate to toxic levels of chlorite. The presence of additional transporters with low affinity for nitrate/chlorate besides NrtA would explain why the deletion of *NRTA* alone did not mediate chlorate resistance.

The partial down-regulation of *NIAD* expression in the Δ*AREA* mutant can be seen in sharp contrast to *A. nidulans* where AreA-homologs were described as obligate activators of all nitrate assimilation genes (Muro-Pastor et al., 1999; Narendja et al., 2002; Berger et al., 2006, 2008). This difference in AreA-dependent regulation between the fungal species is intriguing and is most likely reflected by the genomic localization of the nitrate assimilation genes in the different fungal species. While *NIAD*, *NIIA*, and *NRTA* are clustered in *A. nidulans* (Schinko et al., 2013), they are scattered across different chromosomes in *F. fujikuroi*. It is possible that clustering of genes makes them more accessible to histone rearrangements along the whole genomic region. A possible involvement of AreA in regulating the chromatin accessibility of nitrate assimilation genes in *F. fujikuroi* should be investigated in future studies to elucidate the potential differences in the regulatory mechanisms between the fungal species.

### NirA Is Sorted to the Nucleus in a Nitrate- and NR-Dependent Manner

The gene encoding the pathway-specific TF NirA is weakly expressed under nitrogen-limiting conditions, irrespective of the presence of nitrate in *F. fujikuroi* (**Figure 3A**), similar to the situation in *A. nidulans* (Burger et al., 1991). In addition to transcriptional regulation, NirA in *A. nidulans* was shown to be regulated by a nitrate-, nitrite-, and chlorate-induced nuclear translocation (Berger et al., 2006; Schinko et al., 2013).

Our results suggest a similar regulation of NirA by nucleocytoplasmatic shuttling in *F. fujikuroi*. Under low glutamine conditions, the protein was mainly localized in the cytoplasm due to basal constitutive expression levels of the *NIRA* gene. Upon the availability of nitrate, but surprisingly not nitrite, NirA quickly accumulates in the nucleus (**Figure 4**). We suggest that the nuclear translocation shown to occur within mere seconds allows the immediate transcriptional activation of the main nitrate assimilation genes and a rapid adaption to changing nitrogen conditions. A regulation of NirA abundance via *de novo* protein synthesis would take longer (minutes) due to the time-consuming process of gene transcription and translation (Aymoz et al., 2016).

Our localization studies demonstrated that the nitrate-induced translocation of NirA is independent of the TF AreA and the nitrate transporter NrtA. In contrast, the nuclear accumulation of NirA was significantly less efficient upon addition of nitrate in the *AREA* deletion background in *A. nidulans*, most likely due to the lack of AreA-dependent nitrate transporter activation and the resulting limitation of the nitrate influx (Wang et al., 2008).

Beside the regulation via subcellular shuttling, regulation of the nitrate assimilation pathway is also mediated at the level of transcript stability in *A. nidulans.* For a number of genes, e.g., for *NIAD* and *NIIA*, destabilization of the transcripts in response to glutamine and stabilization of the transcripts in response to nitrate has been demonstrated, which depends on the 3′ UTR of the respective gene (Caddick et al., 2006). However, this mechanism cannot be responsible for the specific accumulation of NirA we observed 60 min after addition of nitrate (**Figure 5**) because the *NIRA::GFP* fusion construct is not terminated by the native *NIRA* terminator but instead by the heterologous glucoamylase terminator of *A. nidulans*

Therefore, the strong accumulation of the NirA protein in a time course of 60 min is likely due to nitrate-induced protein stabilization, e.g., by regulating proteasome-mediated degradation of target proteins via ubiquitination and stabilization through de-ubiquitination (Leach and Brown, 2012). However, the observed quick nuclear accumulation of NirA almost immediately after nitrate addition cannot be explained by *de novo* protein synthesis due to the time-delay between both effects. It is most likely that small amounts of NirA are always present inside the cell (due to the constitutive expression of the *NIRA* locus, **Figure 3**), which are immediately translocated to the nucleus in a short-term response to nitrate. As a second effect, the presence of nitrate induces an overall stabilization of the NirA protein, leading to its accumulation over time and enhancement of its activity. This multi-level regulation of NirA would enable the fungus to quickly adapt to the new conditions and to differentiate between a short- and long-term response to nitrate.

One of the most striking results of our work is the potential role of the NR in nitrate sensing. In contrast to *A. nidulans*, where a ‘pseudo-constitutive’ nuclear localization of NirA was observed in the Δ*NIAD* mutant (Schinko et al., 2013), we did not observe any detectable nuclear accumulation of NirA in the *NIAD* deletion background (**Figure 6**), which is not caused by a general lack of the protein in this strain (**Figure 5B**). This suggests that the NR is able to sense its nitrate substrate and transduce the nitrate signal to NirA. However, despite the fact that we did not see a clear nuclear localization of NirA in the Δ*NIAD* mutant, at least small amounts of NirA must be present inside the nucleus because the expression of the NirA-activated genes in response to nitrate was unaffected in this mutant (**Figure 3**).

### Differences between Nitrate- and Nitrite-Induced Responses

In contrast to nitrate, we neither observed any expression of nitrate/nitrite assimilation genes (**Figure 3B**), nor increased protein levels (**Figure 5**) and nuclear translocation of NirA (**Figure 4**) in response to nitrite. This is surprising, because nitrite, in a similar manner to nitrate and chlorate, was shown to induce expression of *NIAD*, *NIIA*, and *NRTA* in *A. nidulans* and *N. crassa* (Crawford and Arst, 1993; Marzluf, 1997; Berger et al., 2006). Instead, we observed an expression of *NIIA* under nitrogen-starvation conditions and upon the addition of nitrate alone (**Figure 3B**). Despite the non-detectable transcript levels in response to nitrite in *F. fujikuroi*, at least low NiR enzyme levels must be present under this condition, because the fungus is clearly able to grow with nitrite as the sole nitrogen-source (**Figure 1**).

An explanation for the lack of nitrite-induced gene expression may be the toxicity of nitrite which makes it an even less favored nitrogen-source than nitrate. Because of this, many organisms actually have a tightly regulated nitrite uptake and export system to keep intracellular nitrite concentrations below toxic levels (Crawford and Arst, 1993; Lea et al., 2004; Wang et al., 2008; Jia et al., 2009; Cabrera et al., 2014). Taking this into consideration, it could be possible that extracellular nitrite induces only a slight or a significantly delayed expression of the nitrate assimilation system in *F. fujikuroi*, which was not detectable by Northern blot analysis. This would ensure that only limited amounts of nitrite are imported inside the cell, when they are absolutely necessary for growth. Additionally, the observed *NIIA* expression under nitrogen-starvation conditions would ensure that the NiR protein is already present inside the cell before the fungus comes into contact with nitrite as a potential nitrogen source. In this case, the toxic nitrite would be immediately metabolized to glutamine. The elucidation of these nitrite-specific responses in *F. fujikuroi* would be an interesting field for future studies.

### Sensing of Nitrate

In *F. fujikuroi*, it was shown that the expression of many SM gene clusters is subject to NMR, including the GA and BIK biosynthesis genes (Candau et al., 1992; Mihlan et al., 2003; Wiemann et al., 2009, 2013; Tudzynski, 2014). The main effector mediating rapid and strong repression of these two SM clusters was shown to be glutamine, but a possible direct impact of nitrate on their expression needed to be investigated. In this study, we clearly showed that nitrate is not directly sensed as an effector of NMR, and rather that the repression of GA and BIK cluster genes in response to nitrate starts only after its conversion to ammonium and glutamine.

However, the fungus must be able to sense the presence of nitrate when preferred nitrogen sources are absent in order to quickly adapt to the changed conditions, e.g., by the nuclear translocation of NirA and subsequent transcriptional activation of the nitrate assimilation genes. The identification of a responsible nitrate sensor protein mediating the nitrate signal would be very intriguing. Research in *A. nidulans* has provided new insights regarding the signal transduction that leads to the nitrate-responsive nuclear retention of NirA, but the actual sensor still has not been identified (Bernreiter et al., 2007; Schinko et al., 2013; Gallmetzer et al., 2015).

Based on our results, the NR may play a possible nitrate sensing role based on the following reasoning. (1) The nitrate-dependent translocation of NirA was clearly disturbed in the Δ*NIAD* mutant (**Figure 6**). (2) The NR binds nitrate as a substrate, which might result in conformational changes and transduction of the signal to other proteins. The NR could therefore act as a nitrate sensor besides its enzymatic function. We recently described a similar dual function for the *F. fujikuroi* GS, which converts ammonium to glutamine. At the same time, the GS is involved in NMR-dependent regulation by transducing the presence of its substrate, ammonium, to other regulators (Wagner et al., 2013). However, even if the NR plays a role as a nitrate sensor, it cannot be the only one in *F. fujikuroi* because nitrate-induced gene expression was not disturbed in the Δ*NIAD* mutant.

Another interesting observation was that the nitrate-induced transcriptional activation of *NIAD* (**Figure 3**) and nuclear accumulation of NirA (**Figure 6**) occurred in the Δ*NRTA* mutant in a Wt-like manner. At first sight this may appear to be contradictory because our results identified NrtA as the main nitrate transporter in *F. fujikuroi*. However, this contradiction could be explained by three possibilities: (1) Traces of nitrate can be utilized without transporters by passive membrane permeation, (2) other transporters are able to transport low amounts of nitrate inside the cell, which are insufficient for growth but induce regulatory responses, and (3) nitrate is recognized before being internalized by an extracellular sensor protein. It should be noted that nitrate can also be generated inside the cell by oxidation of toxic nitrogen-monoxide (NO) radicals by flavohemoglobins (Schinko et al., 2010, 2013). However, this cannot be the explanation for the nitrate-induced effects we observed in Δ*NRTA*, since they only occurred specifically after the addition of extracellular nitrate. In *A. nidulans* it was shown that the nitrate-induced translocation of NirA was significantly reduced in an *AREA* deletion strain due to the down-regulation of nitrate transporters in this strain. This would indicate that passive nitrate uptake via membrane permeation (Wang et al., 2008) is not sufficient for nitrate-mediated gene activation. Additionally, our NirA-localization studies showed that NirA-translocation in Δ*NRTA* takes place even when very low concentrations of nitrate are added to the cells, making a passive nitrate-uptake by membrane permeation less likely.

This leaves the more plausible possibilities of unspecific nitrate transport by other transporters and/or the presence of a sensor that recognizes extracellular nitrate. There has been no evidence for such a particular sensor in other organisms yet, but there are several examples were nutrient transporters have an additional sensing function, therefore known as transceptors (Lorenz and Heitman, 1998; Donaton et al., 2003; Biswas and Morschhäuser, 2005; Boeckstaens et al., 2008; Teichert et al., 2008; Rubio-Texeira et al., 2012; Van Zeebroeck et al., 2014). Possible candidates for unspecific nitrate transport or extracellular nitrate sensing in *F. fujikuroi* could be the previously mentioned nitrite transporter homolog (FFUJ_03993), which would be an interesting target for future characterization studies.

## Conclusion

The results of our studies provide novel insights to the nitrate assimilation and sensing system in *F. fujikuroi*, uncovering similarities to other species but also some unique features. These findings contribute to the general understanding of alternate nitrogen assimilation pathways in fungal systems, which are connected to infection mechanisms of pathogens and regulation of SM production. We demonstrated that the genes *NIAD*, *NIIA* and *NRTA* encode the sole functional NR, NiR, and nitrate transporter in *F. fujikuroi*, respectively, and that their expression is induced by the presence of nitrate and repressed by high concentrations of the favored nitrogen-source glutamine, in a similar manner to the regulation-pattern in other organisms. In addition, both the pathway-specific TF NirA and the global, nitrogen-dependent regulator AreA act as positive regulators of the nitrate assimilation genes in *F. fujikuroi*, whereas AreB is not involved. While the expression of all genes depends clearly on NirA activity, AreA is essential for transcriptional activation of *NIIA* and *NRTA*, but surprisingly not for expression of *NIAD*. This indicates different regulatory mechanisms in this pathogen compared to other fungal species. In accordance with the findings in *A. nidulans*, NirA itself is regulated post-translationally by a rapid, nitrate-induced nuclear accumulation and increasing levels of NirA protein, probably due to increased protein stability by nitrate. The nuclear translocation apparently depends on the NR, which indicates a potential dual role of the NR as a nitrate sensor. In contrast to nitrate, no nitrite-induced effects were observed on gene expression or NirA-translocation. Therefore, a more detailed study of the nitrite-specific transport and assimilation system in *F. fujikuroi* should be considered.

## Materials and Methods

### Fungal Strains and Culture Conditions

In this study the following *F. fujikuroi* strains were used: Wt strain IMI58289 (Commonwealth Mycological Institute, Kew, UK), Δ*AREA*-T19 (Tudzynski et al., 1999) and Δ*AREB*-T2.1 (Michielse et al., 2014). Strains were maintained on solid CM (Pontecorvo et al., 1953) and cultivated at 28°C in darkness. Cultivation was performed as described in Wiemann et al. (2012), with liquid or solidified synthetic ICI (Imperial Chemical Industries, UK) minimal medium (Geissmann et al., 1966) supplemented with different nitrogen sources at 28°C. For Northern blot analysis, the strains were cultivated in liquid ICI for 72 h, harvested and mycelium was used for extraction of total RNA.

For yeast recombination cloning, *Saccharomyces cerevisiae* strain FGSC9721/FY834 (*MATa his3Δ200 ura3-52 leu2Δ1 lys2Δ202 trplΔ63*) (Winston et al., 1995) was cultivated in 5 ml liquid YPD (pH 5.8, 10 g/l yeast extract, 20 g/l Bacto-Trypton (Difco), 20 g/l glucose) medium overnight at 200 rpm and 30°C. The culture was used to inoculate 50 ml liquid YPD and incubated at 200 rpm and 30°C for 4 to 6 h until an OD 600 nm of ∼1 was reached. The harvested yeast cells were also used for yeast recombination cloning (Colot et al., 2006; Schumacher, 2012).

### Bacterial Strains and Plasmid Construction

*Escherichia coli* strain Top10F’ (Invitrogen, Groningen, Netherlands), cultivated in Lysogeny Broth medium (Sambrook et al., 1989) was used for plasmid propagation and amplification. For creation of the gene replacement vectors pΔNIAD, pΔNIIA, pΔNIRA, and pΔNRTA, flanking regions upstream (5′) and downstream (3′) of *NIAD*, *NIIA*, *NIRA*, and *NRTA* were amplified using the respective 5F/5R and 3F/3R primer pairs, while the hygromycin resistance cassette was amplified from pCSN44 (Staben et al., 1989) using the primer pair hphF/hphR (Supplementary Table S1). All three fragments were cloned into *Eco*RI/*Xho*I-restricted pRS426 (Christianson et al., 1992) by yeast recombinational cloning (see above). For transformation, the gene replacement fragments were amplified from the recombinated vectors by the respective primer pairs 5F/3R (Supplementary Table S1).

For generating the pOE::NrtA vector and the pOE::NirA::GFP fusion vector, full-length clones of *NRTA* and *NIRA* were amplified using the primer-combinations OE-NrtA-F/OE-NrtA-R and NIRA-GFP-F/NIRA-GFP-R, respectively, which contain overlapping sequences homologous to their destination vectors. The *NIRA* PCR product and the *Nco*I-digested plasmid pNAN-OGG (Schumacher, 2012), containing a nourseothricin resistance cassette, and a codon-optimized eGFP (Staben et al., 1989; Pöggeler et al., 2003) under control of the constitutive *A. nidulans oliC* promoter and the *gluc* terminator, were co-transformed into *S. cerevisiae* yielding pOE::NirA::GFP by yeast recombination cloning. In a similar manner the *NRTA* PCR product was transformed into vector pRS426 (Christianson et al., 1992), driven by the strong *gpdA* promoter of *A. nidulans* and a nourseothricin resistance cassette, yielding vector pOE::NrtA.

### Fungal Transformations

Preparation of protoplasts of *F. fujikuroi* was carried out as described by Tudzynski et al. (1996). For deletion of *NIAD*, *NIIA*, *NIRA*, and *NRTA*, the *F. fujikuroi* Wt was transformed with gene replacement fragments derived from pΔNIAD, pΔNIIA, pΔNIRA, and pΔNRTA (see above). Homologous gene-replacement was checked by diagnostic PCR with the respective primer pairs 5DIA-F/hphF, 3DIA-R/hphR, and WT-F/WT-R (Supplementary Table S1). For expression of the NirA::GFP fusion construct, the strains Δ*NIRA*, Δ*NRTA*, Δ*NIAD*, and Δ*AREA* were transformed with 20 μg of the pOE::NirA::GFP vector, yielding strains Δ*NIRA-NIRA::GFP*, Δ*NRTA-NIRA::GFP*, Δ*NIAD-NIRA::GFP*, and Δ*AREA-NIRA::GFP*. For constitutive expression of *NRTA*, 20 μg of pOE::NrtA were transformed into Δ*AREA*, yielding strain Δ*AREA-OE::NRTA*. Genomic integration of the overexpression-constructs was checked by diagnostic PCR with primers ogfp-seqR1/NIRA-WT-F and gpd-dia-for/NrtA-wt-R, respectively (Supplementary Table S1). Transformed protoplasts were regenerated at 28°C in a complete regeneration agar (0.7 M sucrose, 0.5 g/l yeast extract) with 100 μg/ml nourseothricin (Werner Agents, Jena, Germany) or 100 μg/ml hygromycin (Sigma–Aldrich, Taufkirchen, Germany) for 4 to 7 days as specified above.

### PCR

PCR reactions contained 25 ng DNA, 5 pmol of each primer, 200 μM desoxynucleotide tri-phosphates, 1 unit BioTherm DNA polymerase and 1x concentration of BioTherm buffer (Genecraft GmbH, Lüdinghausen, Germany). The reactions were started with 4 min at 94°C, followed by 35 cycles of 1 min per kb of the product at 94°C, 1 min at 56–65°C, 1 min at 70°C, and a final 10 min at 70°C. PCR products were cloned into pCR2.1-TOPO (Invitrogen). Resistance cassettes and eGFP for yeast recombination were amplified with the proofreading Phusion DNA polymerase (Finnzymes, Vantaa, Finland). These reactions contained 25 ng DNA, 5 pmol of each primer, 200 μM desoxynucleotide triphosphates, 1 unit Phusion polymerase and 1x concentration of HF-buffer. The reactions were started with 5 min at 95°C, followed by 35 cycles of 1 min at 94°C, 1 min at 56–65°C, 1 min per kb of the product at 72°C, and a final 10 min at 72°C. All used primers are listed in Supplementary Table S1.

### Nucleic Acid Isolation and Northern Blot Analysis

Lyophilized mycelium was ground into a fine powder and dispersed (in the case of DNA for use in PCR) in extraction buffer as described by Cenis (1992). Plasmid DNA was extracted using the Genomed plasmid extraction kit (Genomed, Löhne, Germany). Total *F. fujikuroi* RNA was isolated using the RNAgents total RNA isolation kit (Promega, Mannheim, Germany). For Northern blot analysis, samples of 20 μg of total RNA were transferred to Hybond-N+ membranes after electrophoresis on a 1% (w/v) agarose gel containing 1% (v/v) formaldehyde. DNA probes were labeled with ^32^P isotopes using the random oligomer-primer method (Sambrook et al., 1989). Hybridizations were carried out overnight at 65°C (Church and Gilbert, 1984). The following probes were used and amplified with the indicated primer combinations (see Supplementary Table S1 for primer sequences): *BIK2* (bik2-F/bik2-R), *CPS/KS* (cps/ks-RT-for/cps/ks-RT-rev), *NIAD* (NIAD-WT-F/NIAD-WT-R), *NIIA* (NIIA-WT-F/NIIA-WT-R), *NIRA* (NIRA-WT-F/NIRA-WT-R), *NRTA* (NRTA-WT-F/NRTA-WT-R).

### Protein Isolation and Western Blot Analysis

Total protein was extracted from freeze-dried mycelium as described before (Todd et al., 2005). Thirty micrograms of total protein extract were used per lane and separated by discontinuous SDS-polyacrylamide gel electrophoresis. The 5% loading gel was adjusted to pH 6.8, while the 8% separation gel was used at pH 8.8. The separation gel was semidry electro-blotted to a nitrocellulose membrane. For detection of the NirA::GFP fusion protein, rabbit polyclonal antibodies to Green Fluorescent Protein coupled to horseradish peroxidase (HRP) conjugates (Miltenyi Biotec GmbH, Bergisch Gladbach, Germany) were used for chemiluminescent detection of the proteins.

### Fluorescence Microscopy

Ten microliters of *F. fujikuroi* mycelium, grown in liquid ICI media (with nitrogen source dependent on the experiment), was directly used for microscopy. GFP-fluorescence and Hoechst staining were observed using a Leica DMRBE microscope (Leica, Wetzlar, Germany) equipped with a high-performance charge-coupled device 12 bit SensiCam (PCO AG, Kehlheim, Germany) and filter set L5 (excitation band-pass filter 480/40, dichromatin mirror 505, suppression band-pass filter 527/30) for GFP. Nuclei were stained with Hoechst 33342 (Sigma–Aldrich, Chemie GmbH, Steinheim, Germany) in a 1:1000 dilution in McIlvaine Buffer pH 7.4 (Kangatharalingam and Ferguson, 1984) and visualized with filter set 49 DAPI shift free (excitation G 365, beam splitter FT 395, emission BP 445/50). Images (optical sections and Z-stacks) were captured with an AxioCam MRm camera and analyzed using the Axiovision Rel 4.8 software package (Zeiss, Germany).

## Author Contributions

AP and BT contributed to the design of the work. AP and JB were involved in data acquisition. AP, JB, and BT were involved in data analysis. AP and BT wrote the manuscript. All authors revised and approved the manuscript.

## Conflict of Interest Statement

The authors declare that the research was conducted in the absence of any commercial or financial relationships that could be construed as a potential conflict of interest.
