# Supplementary material for: Nitrate Assimilation in Fusarium fujikuroi Is Controlled by Multiple Levels of Regulation
Source: Front Microbiol. 2017 Mar 14;8:381. doi: 10.3389/fmicb.2017.00381 (PMC5348485; doi:10.3389/fmicb.2017.00381)
Supplement: Supplementary file 1 [file Data_Sheet_1.DOCX]

Supplementary Material

The molecular network of nitrate sensing and assimilation in *Fusarium fujikuroi*

Andreas Pfannmüller, Jana M. Boysen and Bettina Tudzynski^*^

*** Correspondence:** Bettina Tudzynski**:** Bettina.Tudzynski@uni-muenster.de

# Supplementary Figures


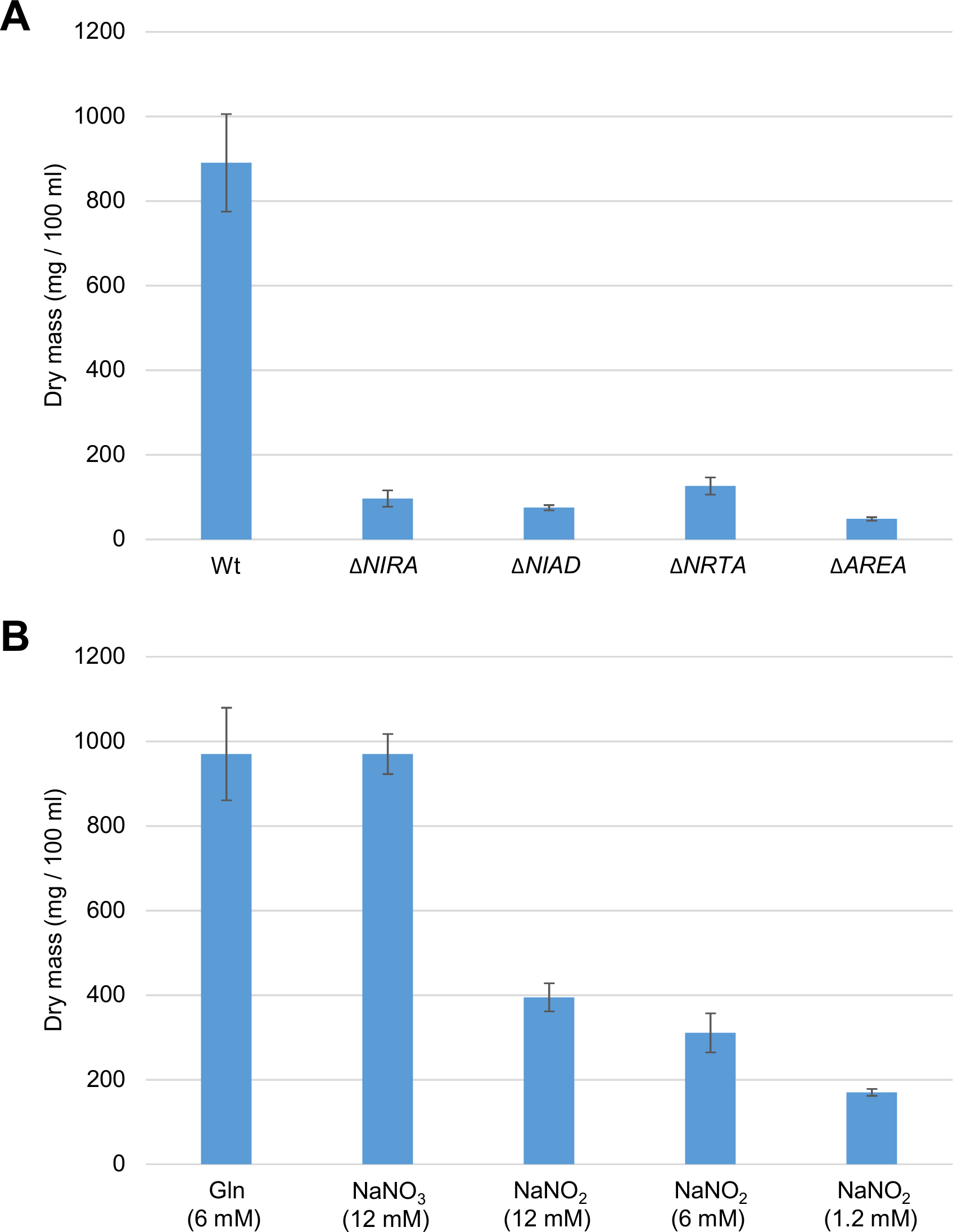


**Supplementary Figure 1.** Biomass formation of *F. fujikuroi* strains in liquid cultures. Strains were cultivated in 100 ml ICI liquid medium supplemented with different nitrogen sources at 28 °C for three days. The depicted mean dry mass of the cultures is based on biological triplicates. Black bars indicate standard deviations. (A) *F. fujikuroi* Wt, Δ*NIRA*, Δ*NIAD*, Δ*NRTA* and Δ*AREA* mutants cultivated with 12 mM sodium nitrate. (B) *F. fujikuroi* Wt cultivated with 6 mM glutamine (Gln), 12 mM sodium nitrate (NaNO_3_) and three different concentrations of sodium nitrite (NaNO_2_).


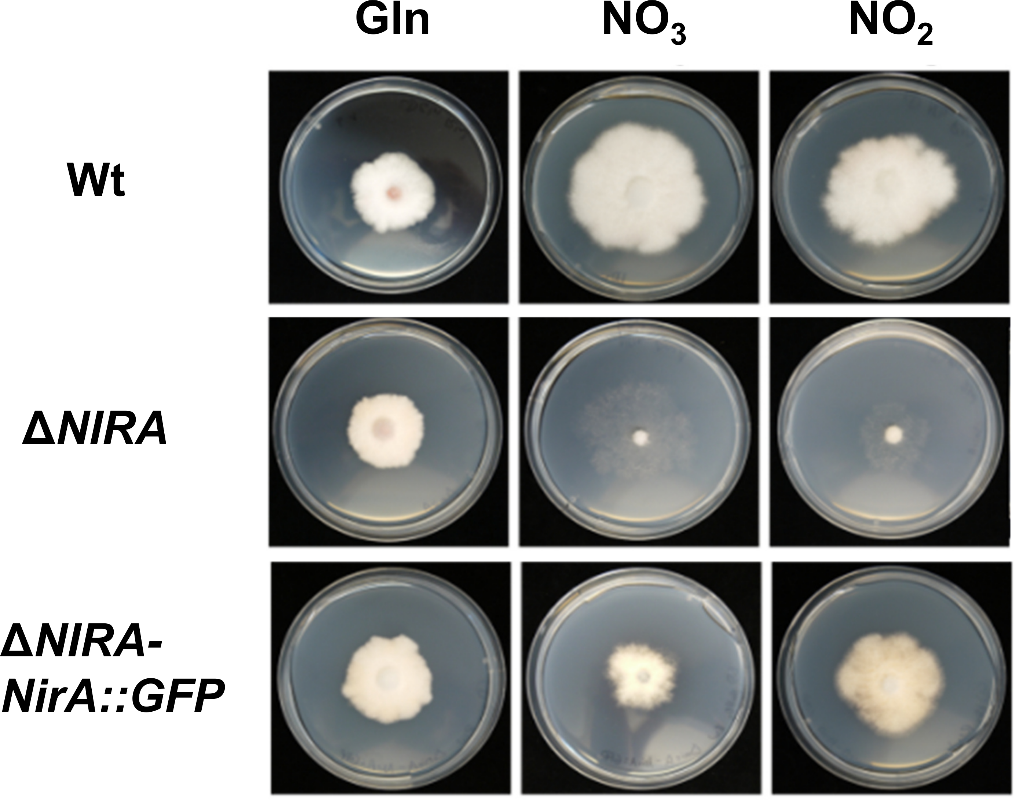


**Supplementary Figure 2.** Expression of the NirA::GFP fusion protein complements the Δ*NIRA* deletion phenotype. The *F. fujikuroi* Wt, the Δ*NIRA* deletion mutant and the Δ*NIAD* mutant transformed with a constitutively expressed NirA::GFP fusion construct were cultivated on solidified synthetic ICI medium supplemented with 6 mM glutamine (Gln), 12 mM sodium nitrate (NO_3_) or 12 mM sodium nitrite (NO_2_) at 28 °C for 4 days.

# Supplementary Tables

| **Supplementary Table 1. Primers used in this study.** | | |
| --- | --- | --- |
| **Name** | **Sequence (5’ – 3’)** | **Usage** |
| bik2-F | CTTGAGTCTGATAGAGGCGC | Probe-generation |
| bik2-R | ACGGCGCAGCAGAAAGTGCC | Probe-generation |
| cps/ks-RT-for | GTGTAGCTGGATCATAGCGACACTCCTG | Probe-generation |
| cps/ks-RT-rev | CCATTGGCCCTGGCTAAGTTTCCC | Probe-generation |
| gpd-dia-fwd | catcttcccatccaagaacc | Diagnostic PCR |
| hphF | GTCGGAGACAGAAGATGATATTGAAGGAGC | Diagnostic PCR |
| hphR | GTTGGAGATTTCAGTAACGTTAAGTGGAT | Diagnostic PCR |
| NIAD-3DIA-R | ACTGCGTCAGTTTACTTTCCGC | Diagnostic PCR |
| NIAD-3F | CTCCTTCAATATCATCTTCTGTCTCTTTGGGCGATGATCTGATGG | Gene knock out |
| NIAD-3R | GCGGATAACAATTTCACACAGGAAACAGCCATAGTGGTGTTGTTCTTGCCC | Gene knock out |
| NIAD-5DIA-F | GCAATTACCAAAGCTAACGCCG | Diagnostic PCR |
| NIAD-5F | GTAACGCCAGGGTTTTCCCAGTCACGACGTAGGATTCAGTCATCGCATCGC | Gene knock out |
| NIAD-5R | ATCCACTTAACGTTACTGAAATCATGTGTGCCAGTTGTGAAGAGG | Gene knock out |
| NIAD-WT-F | AGCCTGAGAAGCAGATCTGC | Diagnostic PCR |
| NIAD-WT-R | AGACGCATCATAAGATGCTGGC | Diagnostic PCR |
| NIIA-3DIA-R | CTTCTCGCTATCGTTTGGTCC | Diagnostic PCR |
| NIIA-3F-F | CTCCTTCAATATCATCTTCTGTCTCCGACTTGGGGATATACCTGAATGGC | Gene knock out |
| NIIA-3F-R | GCGGATAACAATTTCACACAGGAAACAGCAACCAAGTCAGTCCCTTTGCC | Gene knock out |
| NIIA-5DIA-F | AATGCAAGGCAATTCTAGGCC | Diagnostic PCR |
| NIIA-5F-Fwd | GTAACGCCAGGGTTTTCCCAGTCACGACGGACATGACACGAAACTTCGCC | Gene knock out |
| NIIA-5F-Rev | ATCCACTTAACGTTACTGAAATCTCCAACGATAGAAGGGATATCACGGCC | Gene knock out |
| NIIA-WT-F | TGTGGTGGTCGTTGGCTTGGGC | Diagnostic PCR |
| NIIA-WT-R | GCATGCTCGTTCTCAAGTCATCG | Diagnostic PCR |
| NIRA-3DIA-R | AGGCTCCAGCATAAGTCAAAGC | Diagnostic PCR |
| NIRA-3F | CTCCTTCAATATCATCTTCTGTCGGGCTGAATATTCCCGCTACG | Gene knock out |
| NIRA-3R | GCGGATAACAATTTCACACAGGAAACAGCGGCGATTCAATCACTCCAAGC | Gene knock out |
| NIRA-5DIA-F | GTAGCCAATCTGAGGACTCTCC | Diagnostic PCR |
| NIRA-5F | GTAACGCCAGGGTTTTCCCAGTCACGACGCAAAAGGTGAGGAGCCACATCC | Gene knock out |
| NIRA-5R | ATCCACTTAACGTTACTGAAATCTAACTGAGCAAGCGAAGTCTGG | Gene knock out |
| NIRA-GFP-F | CCATCACATCACAATCGATCCAACCGTTACACCATGGAAGAACCGG | GFP fusion |
| NIRA-GFP-R | TACTTACCTCACCCTTGGAAACCATATCTAAGCCTGGAAGCCACCC | GFP fusion |
| NIRA-WT-F | TCTGCAGATGTCAAAGCTTTCCG | Diagnostic PCR |
| NIRA-WT-R | CACTGCGATGTTACAAATCTGGC | Diagnostic PCR |
| NRTA-3DIA-R | AATGTTGAGACGAGTTGGCCC | Diagnostic PCR |
| NRTA-3F | CTCCTTCAATATCATCTTCTGTCTCCGACCACTAAGTGCAGAGTTGCAGC | Gene knock out |
| NRTA-3R | GCGGATAACAATTTCACACAGGAAACAGCATTGCAAAGCCGTCATGTCCG | Gene knock out |
| NRTA-5DIA-F | ACAAAAGCCACCCTATAGCGG | Diagnostic PCR |
| NRTA-5F | GTAACGCCAGGGTTTTCCCAGTCACGACGTTAGCCTTGTGCCCTTATCGC | Gene knock out |
| NRTA-5R | ATCCACTTAACGTTACTGAAATCTCCAACGAAAACCCATCTTGAGCAAGC | Gene knock out |
| NRTA-WT-F | CCTTTCCACCCCTATTGACG | Diagnostic PCR |
| NRTA-WT-R | GCCATGACCACGATGAGGCC | Diagnostic PCR |
| OE-NrtA-F | TACCCCGCTTGAGCAGACATCAAATTCTTTCGTCCGTTCCC | Overexpression |
| OE-NrtA-R | CGGATAACAATTTCACACAGGAAACAGCACAGGTCTCATTCGTGTCCCG | Overexpression |
| ogfp-seqR1 | CGTCTCCCTCACCCTCTCCG | Diagnostic PCR |
